# Growth of Hg_0.7_Cd_0.3_Te on Van Der Waals Mica Substrates via Molecular Beam Epitaxy

**DOI:** 10.3390/molecules29163947

**Published:** 2024-08-21

**Authors:** Shuo Ma, Wenwu Pan, Xiao Sun, Zekai Zhang, Renjie Gu, Lorenzo Faraone, Wen Lei

**Affiliations:** 1 ARC Centre of Excellence for Transformative Meta-Optical Systems (TMOS), Department of Electrical, Electronic and Computer Engineering, The University of Western Australia, Perth, WA 6009, Australia; shuo.ma@research.uwa.edu.au (S.M.); wenwu.pan@uwa.edu.au (W.P.); zekai.zhang@uwa.edu.au (Z.Z.); renjie.gu@uwa.edu.au (R.G.); lorenzo.faraone@uwa.edu.au (L.F.); 2 John de Laeter Center, Curtin University, Bentley, WA 6102, Australia; xiao.sun@curtin.edu.au

**Keywords:** HgCdTe, molecular beam epitaxy, van der Waals, mica substrate

## Abstract

In this paper, we present a study on the direct growth of ${Hg}_{0.7}{Cd}_{0.3}Te$ thin films on layered transparent van der Waals mica (001) substrates through weak interface interaction through molecular beam epitaxy. The preferred orientation for growing ${Hg}_{0.7}{Cd}_{0.3}Te$ on mica (001) substrates is found to be the (111) orientation due to a better lattice match between the ${Hg}_{0.7}{Cd}_{0.3}Te$ layer and the underlying mica substrate. The influence of growth parameters (mainly temperature and Hg flux) on the material quality of epitaxial ${Hg}_{0.7}{Cd}_{0.3}Te$ thin films is studied, and the optimal growth temperature and Hg flux are found to be approximately 190 °C and 4.5 × $10^{-4}$ Torr as evidenced by higher crystalline quality and better surface morphology. ${Hg}_{0.7}{Cd}_{0.3}Te$ thin films (3.5 µm thick) grown under these optimal growth conditions present a full width at half maximum of 345.6 arc sec for the X-ray diffraction rocking curve and a root-mean-square surface roughness of 6 nm. However, a significant number of microtwin defects are observed using cross-sectional transmission electron microscopy, which leads to a relatively high etch pit density (mid-$10^{7}$ ${cm}^{-2}$) in the ${Hg}_{0.7}{Cd}_{0.3}Te$ thin films. These findings not only facilitate the growth of HgCdTe on mica substrates for fabricating curved IR sensors but also contribute to a better understanding of growth of traditional zinc-blende semiconductors on layered substrates.

## 1. Introduction

Infrared (IR) sensing and imaging technology has a number of strategically important applications such as night vision, surveillance, remote sensing, and medical imaging [1]. The critical electronic components for these IR applications are high-performance IR detectors. Among the various IR detector technologies, HgCdTe-based IR detectors have dominated the high-performance end of the IR sensing and imaging market for decades due to their unsurpassed device performance including high detectivity, high quantum efficiency, and fast response time. This is a consequence of their favorable physical properties including a tuneable energy bandgap, a high light absorption coefficient, high carrier mobility, and long minority carrier lifetime [1]. The further development of IR applications requires that future HgCdTe IR detectors have additional features such as lower cost, a larger array format size, higher operating temperature, multiband detection, and a wider field of view (FOV) [2]. Over the past two decades, significant progress has been made in these areas. For example, alternative lattice-mismatched large-area substrates (Si, Ge, GaAs, and GaSb) instead of traditional latticed-matched CdZnTe substrates have been researched for growing HgCdTe IR materials in order to develop detectors with lower cost and a larger array format [3,4,5,6], while novel device structures such as p-i-n, nBn, and fully depleted device structures have been studied for raising the operating temperature [1]. Detector structures such as simultaneous dual color arrays, sequential dual color arrays, and MEMS tuneable filters integrated with the IR detector technology have been developed for multiband/color detection [1].

Regarding the fabrication of IR detectors with a wider FOV, the traditional approach is to use fisheye lenses with complex optics to reduce optical aberrations. However, the use of large, complex, and heavy lenses leads to a reduction in optical signal as well as a large size and weight penalty, which limits their use for new and emerging capabilities such as airborne and field-portable systems [7]. In recent years, metamaterials and their derivative metalenses have been studied with the aim of achieving a wider FOV and reduced aberrations by engineering the physical properties of metamaterials [8]. Although metalenses are of smaller size and lower weight in comparison to the traditional approaches discussed above, they suffer from the limitations of a complex fabrication process, a narrow wavelength band, and low focusing efficiency. Alternatively, curved imaging arrays inspired by the human eye can be used in order to achieve a wider FOV, and eliminating aberrations without adding optical correction elements to the system, and leading to reduced complexity, a smaller size, lower weight, and lower cost [9,10,11]. In recent years, curved CMOS (complementary metal oxide semiconductor) visible imaging sensors have been commercialized by companies such as Curve-One, Sony, Canon, and others [12,13,14]. However, no significant progress has been reported in the area of curved IR imaging arrays, although the US DARPA recently launched the FOCII program to investigate curved IR arrays [15]. The fabrication of curved imaging arrays requires the thinning or removal of epitaxial substrates in order to form the free-standing IR detector array (flip-chip bonded with the readout integrated circuits (ROICs)) which can be curved to the specific radius [16]. Based on current HgCdTe detector processing technology, free-standing IR detector chip thin films could be achieved by means of mechanical grinding combined with ion etching to remove the epitaxial CdZnTe substrates which can, however, cause damage to the HgCdTe thin film materials and degrade device performance. This is due to the fact that HgCdTe materials are much more fragile and sensitive to mechanical damage in comparison to conventional III-V semiconductors, and the material damage caused by polishing and milling can generate substantial crystalline defects that significantly degrade the critical optoelectronic properties such as carrier mobility and minority carrier lifetime and, thus, detector performance. Therefore, one of the key requirements for developing curved IR imaging arrays is to grow high-quality materials and remove the substrate to form free-standing HgCdTe IR detector thin films.

Recent advances in thin film growth technology based on van der Waals epitaxy (vdWE) provides a potential approach for achieving high-quality, large-area, dam-age-free, free-standing HgCdTe epi-layers. The vdWE traditionally defined as the epitaxial growth of van der Waals (vdW) crystals on each other [17] has recently been extended to the epitaxial growth with either the substrate or the epi-layer being layered materials [18]. For vdWE growth, the stringent requirement of lattice–constant matching for high-quality heterostructure growth in traditional semiconductor epitaxy can be relaxed since strong chemical bonding is not present at the interfaces. The weakened vdW interaction at the heterointerface significantly reduces the strain that would typically arise from lattice mismatch in traditional epitaxial growth, thus reducing the generation of misfit dislocations at the heterostructure interfaces [17,18]. Another essential feature of vdWE growth is that the epitaxial layers can be easily lifted off from the substrates to form free-standing thin films due to the weak vdW force between the layers [19]. This enables the easy, damage-free removal of substrates from the epitaxial layers, as required for the fabrication of curved imaging arrays. Although there have been a few reports on vdWE of II-VI semiconductors CdTe [20,21,22], CdSe [19], and ZnTe [23], there is little information on vdWE of HgCdTe in the open literature. A preliminary investigation of the mid-wave IR photoconductors based on HgCdTe grown on mica substrates and the associated lift-off process has been demonstrated in our recent study [24]. It should be noted that mica substrate, a typical layered material that has been widely used for studying vdWE for decades will also be used in this study. In this work, we will present a comprehensive study of the direct growth of ${Hg}_{0.7}{Cd}_{0.3}Te$ materials on mica substrates via molecule beam epitaxy (MBE) and relevant material characterization. The influence of growth parameters on the growth of ${Hg}_{0.7}{Cd}_{0.3}Te$ materials was analyzed in detail, including variations in growth temperature and Hg flux. Various characterization techniques were undertaken to study the material quality of ${Hg}_{0.7}{Cd}_{0.3}Te$ materials directly grown on mica substrates, including high resolution X-ray diffraction (HRXRD), atomic force microscopy (AFM), scanning electron microscopy (SEM), transmission electron microscopy (TEM), and etch pit density (EPD).

## 2. Experimental Results

To better understand the growth of ${Hg}_{0.7}{Cd}_{0.3}Te$ materials on mica substrates, we first studied the influence of growth temperature on material quality. Considering that the optimal temperature window for growing HgCdTe on traditional CdZnTe substrates is very narrow and ranges from 185 °C to 190 °C [1], the temperature for growing ${Hg}_{0.7}{Cd}_{0.3}Te$ materials in this preliminary study varied from 180 °C to 195 °C with other growth parameters being kept constant, including Hg flux (4.5 × $10^{-4}$ Torr), Te flux (1.5 × $10^{-6}$ Torr), and CdTe flux (7 × $10^{-7}$ Torr). The ${Hg}_{0.7}{Cd}_{0.3}Te$ samples (3.5 µm thick) grown at 180 °C, 185 °C, 190 °C, and 195 °C were labeled as Mica001, Mica002, Mica003, and Mica004, respectively.

**Figure 1:**
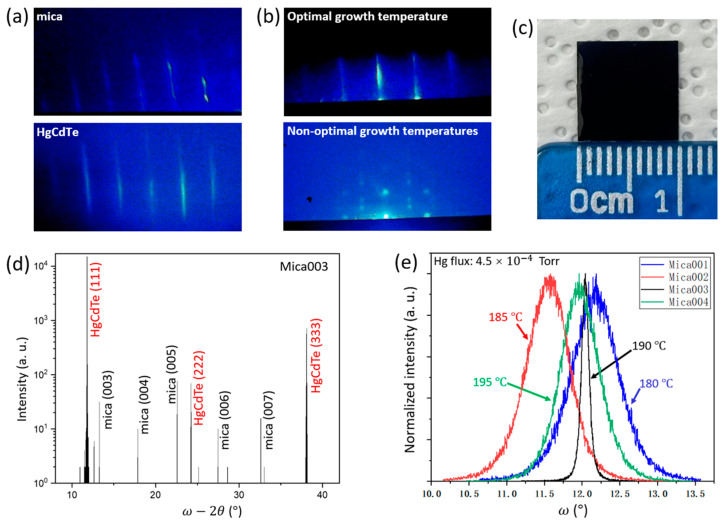
(**a**) Representative RHEED patterns for mica substrate surface just before and after starting ${Hg}_{0.7}{Cd}_{0.3}Te$ growth; (**b**) representative RHEED patterns for ${Hg}_{0.7}{Cd}_{0.3}Te$ surface under an optimal growth temperature (190 °C, sample Mica003) and other non-optimal growth temperatures (180 °C, 185 °C, and 195 °C, samples Mica001, Mica002, and Mica004) at the end of growth run; (**c**) photograph of 3.5 µm thick ${Hg}_{0.7}{Cd}_{0.3}Te$ thin films grown on mica for 2 h and a ruler as reference; (**d**) representative ω−2θ scan HRXRD curve of ${Hg}_{0.7}{Cd}_{0.3}Te$ thin films grown on mica; (**e**) normalized XRD rocking curves of ${Hg}_{0.7}{Cd}_{0.3}Te$ thin films grown on mica at different growth temperatures.

Figure 1a shows the RHEED patterns of the mica substrate after thermal cleaning and before growing HgCdTe, and that of the sample surface right after starting the ${Hg}_{0.7}{Cd}_{0.3}Te$ growth. Obviously, long streak patterns are observed for the sample surface just before and after starting the ${Hg}_{0.7}{Cd}_{0.3}Te$ growth. This indicates that the sample surface has a smooth growth front for growing the subsequent ${Hg}_{0.7}{Cd}_{0.3}Te$ layers, and the initial growth is in a 2D growth mode. However, with the growth of ${Hg}_{0.7}{Cd}_{0.3}Te$ layers, the RHEED patterns evolve differently under different growth temperatures. Under the growth temperature of 190 °C (sample Mica003), the sample surface still presents a long streak RHEED pattern at the end of growth run, as shown in Figure 1b, which indicates the 2D growth mode throughout the growth of ${Hg}_{0.7}{Cd}_{0.3}Te$ layers, and thus high crystalline quality. But under other growth temperatures (180 °C, 185 °C, and 195 °C) the sample surfaces show dotted RHEED patterns, as shown in Figure 1b, which indicates the 3D growth mode at the end of growth run. This suggests the formation of a rough surface and the degraded crystalline quality of the resulting ${Hg}_{0.7}{Cd}_{0.3}Te$ films. Therefore, in this work, 190 °C is labeled to the optimal growth temperature for growing ${Hg}_{0.7}{Cd}_{0.3}Te$ films, while 180 °C, 185 °C, and 195 °C are labeled to the non-optimal growth temperatures for growing ${Hg}_{0.7}{Cd}_{0.3}Te$ films. Figure 1c presents the photograph of a representative ${Hg}_{0.7}{Cd}_{0.3}Te$ sample grown on mica substrate in this work. It is observed that a wafer-scale growth of the ${Hg}_{0.7}{Cd}_{0.3}Te$ epi-layer has been achieved which is crucial for developing curved IR imaging arrays. Figure 1d shows a representative ω−2θ scan HRXRD curve of a ${Hg}_{0.7}{Cd}_{0.3}Te$ thin film grown on mica substrates in this work, with several (00l) peaks observed which represent the crystal planes of single-crystal mica (001) [22]. Apart from the (00l) peaks, the (111), (222), and (333) peaks for the ${Hg}_{0.7}{Cd}_{0.3}Te$ crystal are also observed at around 12.2°, 24.3°, and 38.1°, indicating the out-of-plane growth orientation of ${Hg}_{0.7}{Cd}_{0.3}Te$ grown on mica substrates. The (111) peak also presents a much stronger intensity in comparison to the (222) and (333) peaks. It is worth noting that layered mica substrates have a hexagonal crystal lattice that can better accommodate the growth of zinc blende (ZB) (111) orientation and/or wurtzite (WZ) (0001) since their in-plane crystal lattices are both hexagonal [24,25,26]. This leads to the growth of ${Hg}_{0.7}{Cd}_{0.3}Te$ along the preferred (111) orientation as observed in this work. The in-plane lattice parameters for ZB-${Hg}_{0.7}{Cd}_{0.3}Te$ (111) can be estimated to be a = 4.58 Å and b = 7.93 Å. Since its lattice constant is around 6.47 Å, this indicates that there is a 14% lattice mismatch between the ${Hg}_{0.7}{Cd}_{0.3}Te$ thin film and the fluorphlogopite mica substrate (a = 5.308 Å and b = 9.183 Å) [24]. Figure 1e shows the normalized XRD rocking curves of the four samples, with Mica001, Mica002, Mica003, and Mica004 having an XRD FWHM (full width at half maximum) of 2520 arc sec, 2124 arc sec, 345.6 arc sec, and 1692 arc sec, respectively. Clearly, sample Mica003 presents the smallest XRD FWHM of 345.6 arc sec among all four samples. This result is lower than the published results for the vdWE growth of CdTe on mica substrate [21,22,27] and is comparable to the best reported CdTe grown with vdWE (FWHM ~ 180 arc sec) [20] considering the 3.5 µm thickness of the thin films in this work. However, this FWHM value is 5~6 times larger than that for ${Hg}_{0.7}{Cd}_{0.3}Te$ grown on traditional alternative substrates such as Si, Ge, GaAs, and GaSb that incorporate a CdTe buffer layer [1]. This large FWHM value is beyond the range of our expectations since, in principal, vdWE growth should relax the stringent requirement of lattice–constant matching for high-quality heterostructure growth in traditional semiconductor epitaxy due to the weak vdW force at the interface, and thus result in high-quality heterostructures. The reason for this relatively large XRD FWHM will be discussed later.

**Figure 2:**
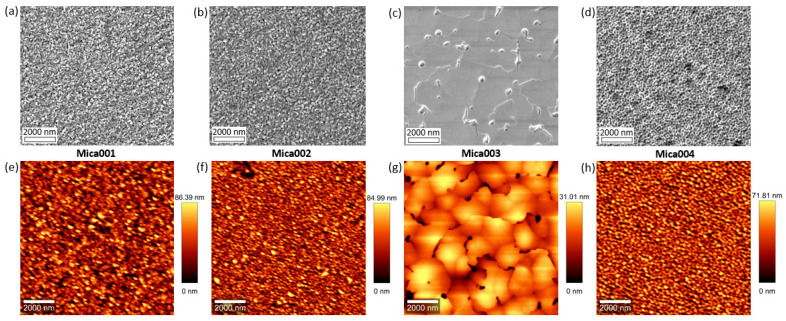
Top-view SEM and AFM images of ${Hg}_{0.7}{Cd}_{0.3}Te$ thin films grown on mica with different growth temperatures: 180 °C (**a**,**e**), 185 °C (**b**,**f**), 190 °C (**c**,**g**), and 195 °C (**d**,**h**), respectively.

Figure 2 shows the SEM and AFM images of all four samples, with the AFM root mean square (RMS) roughness measured to be 18.8 nm, 18 nm, 6 nm, and 18.8 nm for samples Mica001, Mica002, Mica003, and Mica004, respectively. It is also observed that sample Mica003 presents the smoothest surface among the four samples, although some defects and low-angle boundaries are observed on the surface of sample Mica003, the nature of which will be discussed later. For Mica001, Mica002, and Mica004, a high density of defects is observed on the sample surfaces. All these XRD, SEM, and AFM results indicate that 190 °C is close to the optimal temperature for growing ${Hg}_{0.7}{Cd}_{0.3}Te$ on mica substrates, and that the growth window is very narrow. Further work is required in order to fine-tune the growth process to determine the optimum growth temperature within the 185 °C to 195 °C window.

**Figure 3:**
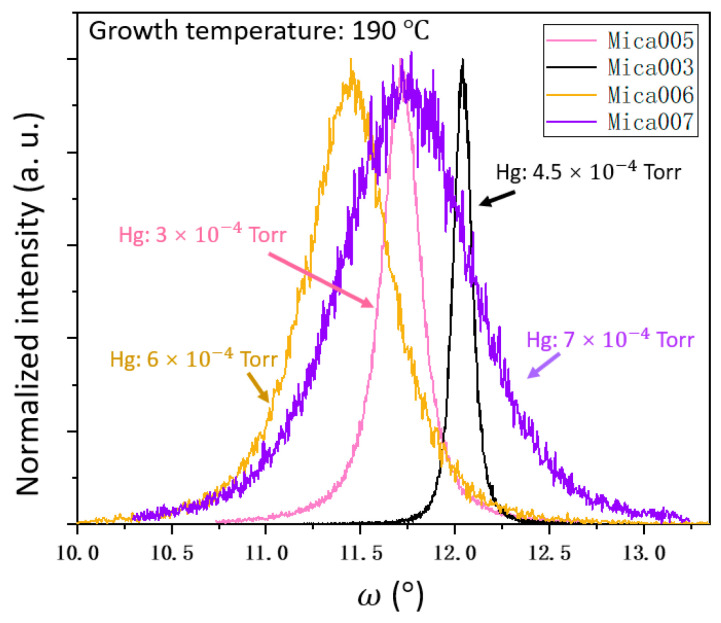
XRD rocking curves of 3.5 µm thick ${Hg}_{0.7}{Cd}_{0.3}Te$ samples grown on mica with different Hg fluxes.

**Figure 4:**
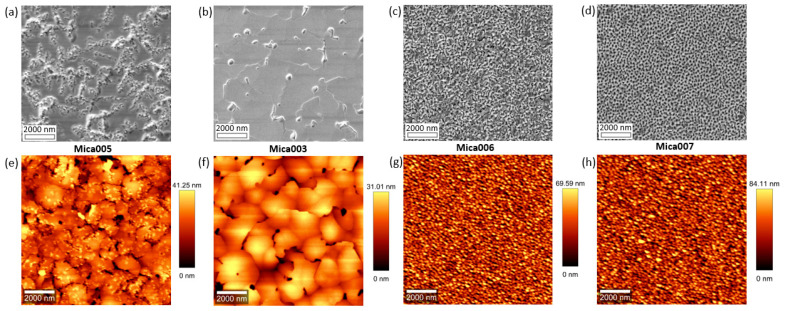
Top-view SEM and AFM images of ${Hg}_{0.7}{Cd}_{0.3}Te$ thin films grown on mica with different Hg fluxes: 3 × $10^{-4}$ Torr (**a**,**e**), 4.5 × $10^{-4}$ (**b**,**f**), 6 × $10^{-4}$ (**c**,**g**), and 7 × $10^{-4}$ (**d**,**h**), respectively.

To better understand the growth of ${Hg}_{0.7}{Cd}_{0.3}Te$ materials on mica substrates, we also studied the influence of Hg flux on the ${Hg}_{0.7}{Cd}_{0.3}Te$ material quality while other growth parameters were kept constant, including the growth temperature (190 °C), Te flux (1.5 × $10^{-6}$ Torr), and CdTe flux (7 × $10^{-7}$ Torr). The ${Hg}_{0.7}{Cd}_{0.3}Te$ samples grown with a Hg flux of 3 × $10^{-4}$ Torr, 4.5 × $10^{-4}$ Torr, 6 × $10^{-4}$ Torr, and 7 × $10^{-4}$ Torr are labeled as Mica005, Mica003, Mica006, and Mica007, respectively. Similarly, XRD, SEM, and AFM were used to characterize the material quality of the ${Hg}_{0.7}{Cd}_{0.3}Te$ samples grown with different Hg fluxes. Figure 3 shows the normalized XRD rocking curves of samples Mica005, Mica003, Mica006, and Mica007, and Figure 4 shows the SEM and AFM images of samples Mica005, Mica003, Mica006, and Mica007. As shown in Figure 3 and Figure 4, samples Mica005, Mica003, Mica006, and Mica007 present an XRD FWHM of 864 arc sec, 345.6 arc sec, 2340 arc sec, and 2880 arc sec, respectively, and an AFM RMS roughness of 8.1 nm, 6 nm, 15.2 nm, and 18.1 nm, respectively. Sample Mica003 is observed to have the smoothest surface in comparison to all other samples. It is worth noting that Mica005 presents an interesting transition feature with a mixture of smooth areas (similar to that of Mica003) and twin-defect areas (similar to that of Mica006 and Mica007). The observation is mainly due to the Hg flux used in this case. For the growth of Hg_0.7_ Cd_0.3_Te, high Hg flux leads to microtwin formation, and low Hg flux leads to poor sample surface roughness. The Hg flux used for growing Mica005 is somewhere in-between, leading to the transition feature observed. By combining the XRD, SEM, and AFM results in Figure 1, Figure 2, Figure 3 and Figure 4, the optimal growth temperature and Hg flux for growing higher quality ${Hg}_{0.7}{Cd}_{0.3}Te$ on mica substrates is observed to be approximately 190 °C and 4.5 × $10^{-4}$ Torr, respectively, which are similar to the reported parameters for the conventional MBE growth of HgCdTe on other non-layered substrates such as CdZnTe, GaAs, Ge, and GaSb, and thus will not be further discussed here [3,6,28]. For a convenient comparison, the samples studied in this work and all parameters are summarized in Table 1.

**Figure 5:**
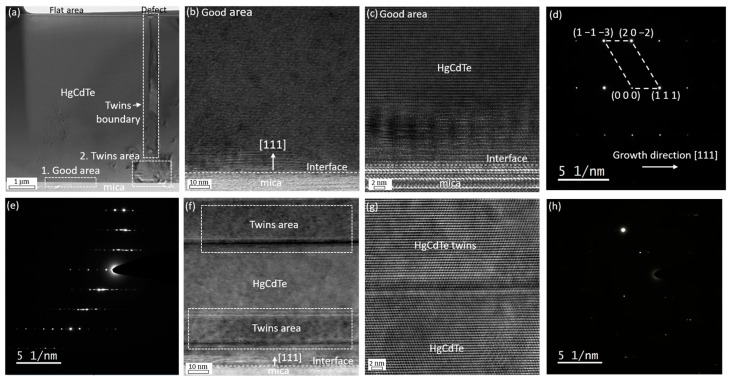
(**a**) Low-magnification cross-sectional TEM image of sample Mica003; (**b**) cross-sectional TEM image and (**c**) HRTEM image of the interface region “1. Good area” as indicated in (**a**); (**d**) SAED patterns of HgCdTe in (**c**); (**e**) SAED patterns of mica in (**c**); (**f**) cross-sectional TEM image and (**g**) HRTEM image of the interface region “2. Twins area” as indicated in (**a**); (**h**) SAED patterns of mica in (**g**).

**Figure 6:**
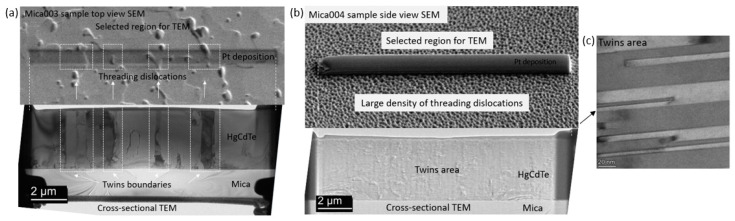
Selected region for TEM specimen preparation and low-magnification cross-sectional TEM image of (**a**) sample Mica003 and (**b**) sample Mica004; (**c**) TEM image of HgCdTe twins area in (**b**).

To better evaluate the crystal quality of ${Hg}_{0.7}{Cd}_{0.3}Te$ materials grown on mica substrates, TEM measurements were undertaken on sample Mica003, and Figure 5 shows the cross-sectional TEM images and SEAD pattern for this sample. As evident in Figure 5a, the cross-sectional TEM image reveals two distinct regions, labeled as “1. Good area” and “2. Twins area”. In the “good” area, a sharp and smooth interface between ${Hg}_{0.7}{Cd}_{0.3}Te$ and mica is observed, as illustrated in Figure 5b,c. The corresponding SAED pattern for ${Hg}_{0.7}{Cd}_{0.3}Te$ and mica (Figure 5d,e) confirms the growth of single-crystal ${Hg}_{0.7}{Cd}_{0.3}Te$ along the [111] direction with a zinc-blende phase on layered mica substrates. In contrast, the “twins” area exhibits microtwins, as depicted in Figure 5f,g. This “rotation” type of twin, also known as lamellar twins, corresponds to a 180° rotation about the [111] growth direction, which involves the presence of one wurtzite bond sequence at the interface [28]. The SAED patterns for the twins area (Figure 5h) displays two sets of diffraction patterns, consistent with previous observations of ${Hg}_{0.7}{Cd}_{0.3}Te$ on mica [24]. These microtwins have been previously reported for HgCdTe grown with a (111) orientation, and the microtwins are observed to be formed more often when growing with (111) growth orientation in comparison to that with conventional (211)B growth orientation [26,28]. As shown in Figure 5f, the microtwins stacking along the (111) orientation can occur multiple times during the growth due to the low energy required to form such a stacking fault [28]. This process eventually results in the formation of twin boundaries and defects observed on the sample surface, as shown in Figure 5a. These defects are likely screw threading dislocations caused by the interaction of microtwin stacking faults and partial dislocations in the twining plane along the growth orientation [29,30]. To further investigate these defects, TEM measurements were also conducted on sample Mica004 for comparison. Figure 6a,b illustrate the selected area for TEM specimen preparation and the cross-sectional TEM images for sample Mica003 and Mica004, respectively. For sample Mica003, as evident from Figure 6a, the twin boundaries extend all the way from the epi-layer/substrate interface to the sample surface, leading to the observed threading dislocations in the region selected for TEM specimen preparation. In contrast, the cross-sectional TEM image of sample Mica004 (Figure 6b) reveals a structure completely composed of microtwins areas (the TEM image of the typical microtwins area is shown in Figure 6c). The increased density of twin boundaries correlates with a higher density of threading dislocations on the sample surface, consistent with the findings on defect formation during the growth of CdTe (111) [29]. This eventually results in degraded crystal quality, poor surface morphology, and increased surface roughness for samples grown under non-optimal growth conditions.

**Figure 7:**
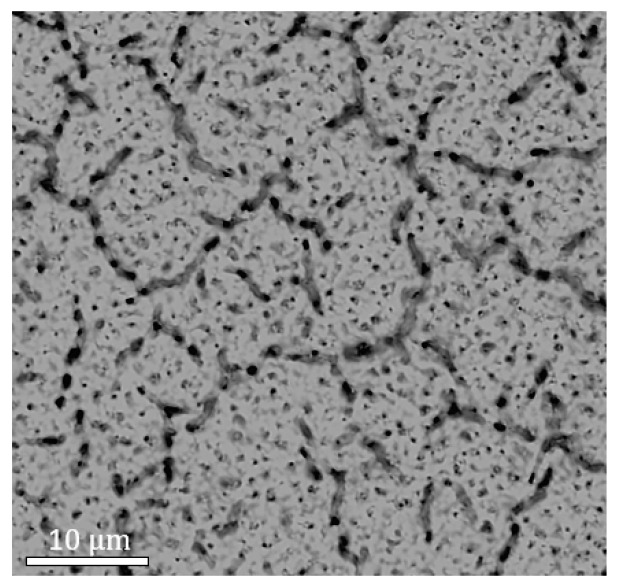
Surface SEM image of sample Mica003 after EPD etching.

Apart from TEM measurements, EPD measurement was also undertaken on sample Mica003 to further evaluate the crystal quality of the ${Hg}_{0.7}{Cd}_{0.3}Te$ materials grown. A standard etchant was used to reveal the EPD of the ${Hg}_{0.7}{Cd}_{0.3}Te$ materials [31]. Figure 7 shows the SEM image after etching, and the EPD was determined to be ~$5\times 10^{7}{cm}^{-2}$. This high EPD value is beyond what was expected, since theoretically vdWE growth should effectively relax the stringent requirement of lattice–constant matching for high-quality heterostructure growth in traditional semiconductor epitaxy due to the weak vdW force at the interface, and thus should reduce the generation of misfit dislocations at the heterostructure interface [17]. However, when combined with the TEM results, this high EPD value is better understood. As shown in Figure 5, these twin boundaries are formed due to the zinc-blende/wurtzite (ZB/WZ) stacking faults, and these twin boundaries can extend all the way from the epi-layer/substrate interface to the sample surface, resulting in the pits observed on the surface. Clearly, these defects contribute to the high EPD numbers, the large XRD FWHM, and the large RMS surface roughness, as observed in Figure 1, Figure 2, Figure 3 and Figure 4.

## 3. Discussion

From the above discussion, it can be summarized that the optimum MBE growth parameters, especially growth temperature and Hg flux, for the vdW growth of ${Hg}_{0.7}{Cd}_{0.3}Te$ on layered mica substrates are similar to those for growing HgCdTe on conventional bulk substrates such as CdZnTe, Si, GaAs, Ge, and GaSb. However, for the best growth conditions determined in this preliminary study, the crystal quality, surface morphology, and EPD values of ${Hg}_{0.7}{Cd}_{0.3}Te$ thin films grown on mica substrates are not as high-quality as theoretically expected for vdW growth. The main reason for this is the generation and formation of twin defects in the ${Hg}_{0.7}{Cd}_{0.3}Te$ materials, which eventually lead to twin boundaries in the ${Hg}_{0.7}{Cd}_{0.3}Te$ materials, the observed pits on the surface, and the resulting high EPD values. Therefore, it is essential to suppress and annihilate the generation and propagation of twin defects in the HgCdTe materials in order to achieve high-quality vdW growth of epi-layers on mica substrates. There are several challenges to be addressed in order to suppress and annihilate the twin defects in HgCdTe materials. They are as follows: (1) Better preparation of substrate surfaces. Mica substrates have relatively low thermal conductivity in comparison to other substrates [32], which could lead to a non-uniform thermal cleaning of the mica substrate surface, and thus non-ideal MBE vdW growth. Thus, it is essential to have better preparation of the substrate surface. (2) Optimized MBE growth conditions. The twin defect formation is very sensitive to growth conditions [28], such as growth temperature and element flux. Although this paper presents a preliminary study on the influence of growth temperature and Hg flux on the material quality of ${Hg}_{0.7}{Cd}_{0.3}Te$, more effort is needed in order to determine the optimal growth conditions to suppress and annihilate twin defects in HgCdTe materials. (3) Buffer layers and/or dislocation filtering layers. Buffer layers and dislocation filtering layers have been widely and successfully used for suppressing and annihilating defects during epitaxial growth of semiconductor heterostructures such as CdTe and HgCdTe on GaAs and GaSb [33,34], which should also be evaluated for growing HgCdTe on mica substrates. All of these issues represent topics for further study in the future.

## 4. Materials and Methods

${Hg}_{0.7}{Cd}_{0.3}Te$ thin film materials were grown on layered fluorphlogopite mica $\left[{KMg}_{3}({AlSi}_{3}O_{10})F_{2}\right]$ (001) substrates in a Riber 32P MBE system equipped with elementary Hg, Te, and compound CdTe as source materials. Mica substrates were prepared by means of mechanical exfoliation to obtain freshly cleaved surfaces in nitrogen atmosphere. The substrates were then mounted with colloidal graphite on molybdenum blocks and loaded into the MBE system. The mica substrates were thermally baked at 150 °C for 60 min under vacuum in the MBE loading chamber to remove water vapor and other volatile contaminants. The prepared mica substrates were then transferred to the growth chamber and thermally cleaned at 400 °C for 40 min, at which stage they were ready for the subsequent epitaxial growth of a 3.5 µm thick ${Hg}_{0.7}{Cd}_{0.3}Te$ thin film. To better understand the growth of ${Hg}_{0.7}{Cd}_{0.3}Te$ on mica substrates, two main growth parameters were studied: growth temperature and Hg flux (beam equivalent pressure). The growth of ${Hg}_{0.7}{Cd}_{0.3}Te$ was completed with a 100 nm thick CdTe capping layer for protection and passivation purposes.

The crystal quality of ${Hg}_{0.7}{Cd}_{0.3}Te$ films was characterized with a PANalytical Empyrean HRXRD instrument. The surface roughness and morphology of ${Hg}_{0.7}{Cd}_{0.3}Te$ were characterized with atomic force microscopy (AFM) (WITEC alpha 300RA+) in tapping mode and scanning electron microscopy (SEM) (Tescan LYRA3 GM). The specimen for cross-sectional transmission electron microscopy (TEM) was prepared using a focused ion beam facility (Tescan LYRA3 GM). The TEM and selected area electron diffraction (SAED) measurements were conducted using a FEI Talos FS200X and a FEI Titan G2 80-200 instrument. The dislocation density of the ${Hg}_{0.7}{Cd}_{0.3}Te$ layer was characterized with conventional EPD measurement [3].

## 5. Conclusions

In summary, this work presents a study on the direct vdW growth of ${Hg}_{0.7}{Cd}_{0.3}Te$ thin films on layered mica (001) substrates with molecular beam epitaxy. The preferred growth orientation is found to be the (111) orientation due to a better matched crystal lattice. The influence of growth parameters (mainly growth temperature and Hg flux) on the material quality of ${Hg}_{0.7}{Cd}_{0.3}Te$ thin films is studied, and the optimal growth temperature and Hg flux are found to be approximately 190 °C and 4.5 × $10^{-4}$ Torr in terms of better crystal quality and surface morphology. ${Hg}_{0.7}{Cd}_{0.3}Te$ thin films grown with the close-to optimal growth temperature and Hg flux present an XRD FWHM of 345.6 arc sec and an RMS surface roughness of 6 nm. However, microtwin defects are observed with cross-sectional TEM. This leads to a relatively high EPD value of mid-10^7^ ${cm}^{-2}$ in the ${Hg}_{0.7}{Cd}_{0.3}Te$ thin films. Some further work is needed in the future to suppress and annihilate the generation and propagation of these twin defects in order to achieve better crystal quality and lower defect density. The findings of this study not only facilitate the growth of HgCdTe on mica substrates for fabricating curved IR sensors but also provide a better understanding of the van der Waals epitaxy growth of traditional zinc-blende semiconductors on layered substrates with molecular beam epitaxy.

## Figures and Tables

**Table 1:** Samples studied in this work with all parameters including growth temperature, flux ratio, XRD FWHM, and RMS roughness.

Sample	Growth Temperature (°C)	Hg Flux (Torr)	Flux (Torr)	FWHM (arc sec)	RMS Roughness (nm)
Mica001	180	4.5 × $10^{-4}$	Te: 1.5 × $10^{-6}$ CdTe: 7 × $10^{-7}$	2520	18.8
Mica002	185	4.5 × $10^{-4}$		2124	18
Mica003	190	4.5 × $10^{-4}$		345.6	6
Mica004	195	4.5 × $10^{-4}$		1692	18.8
Mica005	190	3 × $10^{-4}$		864	8.1
Mica006	190	6 × $10^{-4}$		2340	15.2
Mica007	190	7 × $10^{-4}$		2880	18.1

## Data Availability

Data are contained within the article.
